# Gene-based bin analysis of genome-wide association studies

**DOI:** 10.1186/1753-6561-2-s4-s6

**Published:** 2008-12-17

**Authors:** Nicolas Omont, Karl Forner, Marc Lamarine, Gwendal Martin, François Képès, Jérôme Wojcik

**Affiliations:** 1 Merck Serono International S.A., 9 chemin des Mines, 1202 Geneva, Switzerland; 2 Epigenomics Project, Genopole^®^, 523 Terrasses de l'Agora, 91034 Évry cedex, France

## Abstract

**Background:**

With the improvement of genotyping technologies and the exponentially growing number of available markers, case-control genome-wide association studies promise to be a key tool for investigation of complex diseases. However new analytical methods have to be developed to face the problems induced by this data scale-up, such as statistical multiple testing, data quality control and computational tractability.

**Results:**

We present a novel method to analyze genome-wide association studies results. The algorithm is based on a Bayesian model that integrates genotyping errors and genomic structure dependencies. *p*-values are assigned to genomic regions termed bins, which are defined from a gene-biased partitioning of the genome, and the false-discovery rate is estimated. We have applied this algorithm to data coming from three genome-wide association studies of Multiple Sclerosis.

**Conclusion:**

The method practically overcomes the scale-up problems and permits to identify new putative regions statistically associated with the disease.

## Background

The last years have shown a tremendous increase in the number of markers available for association studies. Previous studies were dealing either with the whole genome at a very low resolution (for instance 5 264 microsatellites in [1]) or with a carefully chosen region of few millions of base pairs [2,3]. Recent technologies allow the genome-wide genotyping of hundred of thousands SNPs [4]. This has arisen the need of new methodological developments to overcome different issues, such as the multiple-testing problem, gene biases, data quality analysis and the computational tractability.

Firstly, the multiple testing problem seems to cause association studies ability to detect associations to decrease as the number of markers increases. The classical analysis strategy, based on an association test for each marker [5], encounters increasing difficulties as more than one million of markers are available: Increasing the number of markers prevents from the detection of the mild genetic effects expected in complex diseases, as only strong effects emerges from the huge noise generated by the increased quantity of data.

Methods like False Discovery Rate (FDR) [6] computation allow to control the error rigorously, but do not increase the statistical power. Better strategies based on haplotype blocks are being developed, the first step being gathering such block data (see the HapMap project, [7]). The gain of such strategies is two-folded: (i) the number of tests is independent of the number of markers (ii) the statistical power may be increased if markers of the same haplotype block are not fully correlated.

Secondly, a genetic association of a given SNP is a statistical feature and does not explain by itself a phenotype. To biologically interpret an associated marker, its haplotype block should first be delimited. Then, the association can be refined by fine-scale genotyping technologies or ideally by full resequencing. This eventually allows to identify functional mutations. Most of the time, these mutations impact relatively close genes. This is a first argument to bias association analysis towards genes. Moreover, even if haplotype blocks are unreachable, DNA might be cut into distinct regions (called *bins*) on another basis, so as to limit the multiple-testing problem and make it independent of the number of markers. Combining these two arguments leads to choose one bin for each gene, and to create "desert" bins in large unannotated regions. It allows to associate a list of genes with a test, which simplifies the analysis of results. The drawbacks are (*i*) that it makes more difficult the study of these "deserts", however the goal is here to maximize, not the chance of finding an association, but the chance of elucidating a mechanism of a complex disease given the current knowledge (*ii*) that a bin might contain several haplotype blocks, resulting in a dilution of the association signal if only one block is associated. Reciprocally, neighbor bins are not independent because they may share a haplotype block. However, with the classical strategy, correlated neighbor SNPs would also be tested separately.

Thirdly, genome-wide genotyping data are obtained by high-throughput experiments which encompass limitations requiring careful statistical methodology. Especially, with *Affy. technology*, the trade-off between the call rate (i.e. errors detected by the genotyping process and resulting in missing genotypes in the data set) and the error rate (i.e. errors left in the data) is difficult to adjust. Obtaining unbiased statistical results is then conditioned to good pre-processing filters. Indeed spurious markers must be eliminated and missing data correctly managed.

In addition, for most of SNPs used in this study, some genotypes are held by less than few percents of patients, which, given the usual collection size of a few hundreds, (*i*) is not enough for good asymptotic approximations and (*ii*) should be considered with care given possible high error rate.

Finally, whatever algorithmic solution is developed, because the number of markers available will probably quickly reach a few millions, creating a scalability problem, it has to be linear in the number of markers.

In this paper we present a novel Bayesian algorithm developed to easily analyze genome-wide association studies. This algorithm is based on a gene-based partitioning of DNA into regions, called bins. A *p*-value of association is computed for each bin. The model takes into account genotyping errors and missing data and tries to detect simple differences in the haplotype block structure between cases and controls. The study of different collections is allowed. The multiple testing problem is addressed by estimation of FDR. The method has been applied to analyze the results of three genome-wide case-control association studies of the complex disease Multiple Sclerosis (MS). It identifies putatively associated bins, containing genes previously described to be linked to MS (see [8] for review) as well as new candidate genes.

## Materials

Three association studies dealing with Multiple Sclerosis (MS) in three independent collections have been realized. Around 600 patients have been recruited for each study, half of them as cases affected by the disease, half of them as controls (Table 1). Genotypes of the 116 204 SNPs have been determined for each patient using Affymetrix GeneChip^® ^human mapping 100 K technology (*Affy. technology*).

**Table 1:** Genome-wide association multiple sclerosis collections.

*Coll.*	*Origin*	*#Cases*	*#Controls*	*%Females*
A	French	314	352	69

B	Swedish	279	301	71

C	American	289	289	85

## Methods

### Notations

Stochastic variables are noted with a round letter (V), a realization is noted in lower case (*v*). Indices are noted in lower case (*k*), ranging from 1 to the corresponding upper case letter (*K*). Unless needed, this range of indices (*k *∈ [1, *K*]) is omitted. The number of different values is noted #(V). The *n*-dimensional table of the number of individuals having the same combination of values for given variables $V^{k}$, *k *∈ [1, *K*] (the contingency table) is noted $n(V^{1},...,V^{K})$. The marginalization of such a contingency table over one variable, for example $V^{1}$, is noted $n(⊕,V^{2},...,V^{K})=\sum_{v\in \#(V^{1})}n,(v,V^{2},...,V^{K})$. Estimation of a probability distribution *P*(V) is noted with hatted letter, $\hat{P}(V)$. Each bin *b *∈ [1, *B*] contains *J*_*b *_genetic markers $G_{b}^{j}$ with *j *∈ [1, *J*_*b*_]. Each patient *i *∈ [1, *I*] has a phenotype value *s*(*i*) (in case-control studies, #(S) = 2), discrete co-variable values *v*_*m*_(*i*), *m *∈ [1, *M*] (gender: *m *= 1, or collection of origin: *m *= 2), and a genotype value for each marker $g_{b}^{j}(i)$ (with SNPs, #($G_{b}^{j}$) = 3). A patient *i *is represented by this vector:

(1) $$i=\left[s(i),v_{m}(i),g_{b}^{j}(i)\right]\text{with}:m\in [1,M],b\in [1,B],j\in [1,J_{b}]$$

The data set is noted *D *= {*i*}_*i *∈ [1, *I*]_. A first level of the method aggregates predictors at the bin level. The "restriction" of a patient to a bin is noted *i*_*b*_, the corresponding data set being *D*_*b *_= {*i*_*b*_}_*i *∈ [1, *I*]_:

(2) $$i_{b}=\left[s(i),v_{m}(i),g_{b}^{j}(i)\right]\text{with}:m\in [1,M],j\in [1,J_{b}]$$

### Data preprocessing

Due to *Affy. technology *(the D.M. calling algorithm), errors on heterozygotic genotypes are more frequent. It can be detected through the deviation of a SNP from the Hardy-Weinberg equilibrium, which basically states that, noting *P*(*a*) = *P*(*aa*) + *P*(*Aa*)/2 and *P*(*A*) = *P*(*AA*) + *P*(*Aa*)/2:

(3) $$\{\begin{array}{l} P(aa)=P{\left(a\right)}^{2}\\ P(Aa)=2P(a)P(A)\\ P(AA)=P{\left(A\right)}^{2} \end{array}$$

Therefore, the following pre-processing filters are applied: SNPs are discarded (*i*) if the number of missing genotypes is higher than 5% because the genotyping process quality was low for this SNP, (*ii*) if the minimum allele frequency in controls MAF = min(*P*(*a*), *P*(*A*)) is lower than 1%, because the SNP holds no information, or (*iii*) if the probability that the SNP follows the Hardy-Weinberg equilibrium in controls is lower than 0.02.

### Bin definition

**Figure 1:**
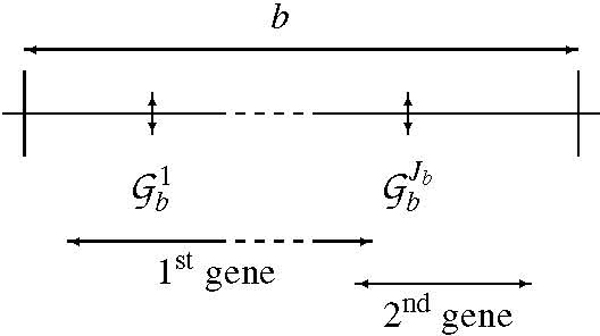
Representation of a bin containing two genes and *J*_*b *_markers.

Bins are defined on DNA from protein genes as defined in the version 35.35 of EnsEMBL [9] of the human DNA sequence. The basic region of a gene lie from the beginning of its first exon to the end of its last exon. Overlapping genes are clustered in the same bin. If two consecutive genes or clusters of overlapping genes are separated by less than 200 kbp, the bin limit is fixed in the middle of the interval. Otherwise, the limit of the upstream bin is set 50 kbp downstream its last exon, the limit of the downstream bin is set 50 kbp upstream its first exon, and a special bin corresponding to a *desert *is created in between the two bins. With these rules, desert bins have a minimum length of 100 kbp (Figure 1).

### Assessing bin association

#### General model, hypotheses and statistics

We assume that each bin constitutes an independent data set. The following ideal probability distribution is defined:

(4) $$\forall b\in [1,B],P(I_{b})=P(S,V_{m},G_{b}^{j})$$

As experimenters choose cases and controls (phenotypes) each individual subset of the study is a realization of the conditional distributions $P(G_{b}^{j}|S,V_{m})$. Estimations of probability distribution are possible from contingency tables:

(5) $$\hat{P}(G_{b}^{j}|S,V_{m})=\frac{n(S,V_{m},G_{b}^{j})}{n(S,V_{m},⊕)}$$

On the contrary, due to the experimental design, estimations of $P(S,V_{m})$ are impossible.

A general way to assess the association of a bin b is to estimate whether ${\left(G_{b}^{j}\right)}_{j\in [1,J_{b}]}$ is independent from the phenotype S, i.e., whether $P(G_{b}^{j}|V_{m})$ is "far" from $P(G_{b}^{j}|S,V_{m})$.

(6) $$H_{0}^{b}:P(G_{b}^{j}|S,V_{m})=P(G_{b}^{j}|V_{m})$$

However, as only $P(G_{b}^{i}|V_{m},S)$ is estimable, estimation of $P(G_{b}^{j}|V_{m})$ is not possible. Therefore, one estimates $\hat{P_{H_{0}^{b}}}(G_{b}^{j}|V_{m})$ assuming $H_{b}^{0}$, as indicated by the subscript:

(7) $$\hat{P_{H_{0}^{b}}}(G_{b}^{j}|V_{m})=\frac{n(⊕,V_{m},G_{b}^{j})}{n(⊕,V_{m},⊕)}$$

We have chosen likelihood ratio LR as a statistic to estimate the "distance" between estimations of $P(G_{b}^{j}|S,V_{m})$ and $P(G_{b}^{j}|V_{m})$. For each patient, the LR is:

(8) $$\text{LR}(i_{b})=\frac{\hat{p}(g_{b}^{j}(i)|s(i),v_{m}(i))}{\hat{p_{H_{0}^{b}}}(g_{b}^{j}(i)|v_{m}(i))}$$

As all patients are considered to be independently chosen, the LR of the set of patients available is:

(9) $$\text{LR}(D_{b})=\prod_{i\in [1,I]}\text{LR}(i_{b})$$

#### *p*-value estimation and FDR

To assess estimation errors due to randomness and sample size, the probability that $H_{b}^{0}$ is true given the observation, i.e. the *p*-value *π*_*b *_needs to be computed. This is theoretically achieved by enumerating all possible outcomes *D*_*b*_(*σ*) of the experiment that lead to the observed data *D*_*b*_(*σ*_0_) (*σ *is a enumeration parameter to be defined. The following notation simplification is done: *D*_*b*_(*σ*_0_) = *D*_*b*_). Then the probability *p*(*D*_*b *_(*σ*)) of each outcome assuming that $H_{b}^{0}$ is true is computed as well as its LR. Finally, the *p*-value is:

(10) $$\begin{array}{c} \pi_{b}=p(\text{LR}(D_{b}(\sigma ))\geq \text{LR}(D_{b}))\\ =\sum_{\sigma |\text{LR}(D_{b}(\sigma ))\geq \text{LR}(D_{b})}p(D_{b}(\sigma )) \end{array}$$

In this article, estimation of *p*-values is based on permutations: possible outcomes are obtained through patient phenotype permutations *σ *and *σ*_0 _is the identity permutation. The probability of each permutation is uniform. The denominator of equation (8) is constant with respect to such permutations, therefore it is omitted. Sampling this space is possible: random permutations of the phenotypes are drawn and used to compute a LR. This is a Monte-Carlo procedure, for which we propose an optimized implementation that guarantees the precision required for FDR estimation:

For each bin *b*, compute LR for new permutations of phenotypes until the number of permutations realized *N*_*b *_satisfies the following equation, noting ${\hat{\pi }}_{b}$ the estimation of the bin *p*-value:

(11) $$N_{b}\geq {\left(B\theta \frac{\gamma }{\delta }\right)}^{2}\min\left(\frac{1-\theta }{\theta },\frac{1-{\hat{\pi }}_{b}}{{\hat{\pi }}_{b}}\right)$$

*θ *and *γ*/*δ *control the quality of the method: *θ *is an upper bound of the threshold that is expected to be used to select bins. *γ*/*δ *controls the error due to the randomness of the process: Assuming that two consecutive *p*-values *π*_*b*1 _<*π*_*b*2 _≈ *θ *are sufficiently spaced (probability *p*_*s *_= *e*^-*δ*^), ${\hat{\pi }}_{b1}<{\hat{\pi }}_{b2}$ with a confidence *c *= cdf(N(0, 1), *γ*) (standard normal cumulative distribution function). In this article, *B *= 11 264, *θ *= 0.001, *δ *= 1 and *γ *= $\sqrt{2}$ thus *N*_*b *_= 507 003, *p*_*s *_= 0.37 and *c *= 0.92.

To address multiple testing, the method uses an FDR estimation defined as in [10]:

(12) $$\text{FDR}(\theta )=\frac{\hat{\Pi_{0}}\theta B}{\#(\{b|\pi_{b}<\theta \})}$$

The numerator is an estimation of the expectation of the number of false-positive with *π*_*b *_≤ *θ*. $\hat{\Pi_{0}}$ is an estimation of the proportion of bins under the null hypothesis. Given that it is expected to be very high in current study, it is (conservatively) fixed at its upper bound: $\hat{\Pi_{0}}$ = 1. The denominator is the number of tests with *p*-values below. The ratio is therefore an estimation of the proportion of false negatives in the set of bins with a *p*-value below *θ*. Because we want to analyze thoroughly the FDR for around the 10 bins with the lowest *p*-values, the FDR is not controlled at a specified threshold as in [6] but only estimated.

This estimation relies on two main hypothesis: (*i*) tests are independent or positively correlated [11], (*ii*) *p*-values are continuously and uniformly distributed in [0, 1]. Assuming that sharing of haplotype block by neighbor bins is the only source of correlation between tests, the positive correlation seems reasonable. Indeed, if the *p*-value of a not associated bin decreases, the *p*-values of bins sharing the same haplotype block are more than likely to decrease too. The uniform distribution is less obvious, because the number of possible contingency tables is finite so that even the null distribution is not uniform. However, the sample size is one to two order of magnitude higher than in other applications of FDR to discrete data in which the problem is acute [12].

#### Model of linkage disequilibrium and error

Correlation between markers induced by LD is modelled with an inhomogeneous hidden Markov chain of order 1. Indeed, as a rough approximation, for each marker, most information is found on its first neighbor on each direction of DNA. In a directed graphical model, independence assumptions consist in:

(13) $$P{\left(G_{b}^{j}|G_{b}^{l}\right)}_{l\neq j}=\{\begin{array}{ll} P\left(G_{b}^{j}|G_{b}^{j-1}\right) & \text{if }j\neq 1\\ P\left(G_{b}^{j}\right) & \text{if }j=1 \end{array}$$

Finally, this assumptions also allow to obtain correct estimations because corresponding contingency tables are sufficiently filled. They implies that contingency tables are computed for 2 SNPs (#($G_{b}^{j}$) = 3), the phenotype (#(S) = 2) and the co-variables together. The gender co-variable is not be used. It requires the hypothesis that the SNP distribution is independent from it. The only co-variable is the study patients belong to (Table 1, #($V_{2}$) = 3). As collection sizes for a given study are around 600, the average number of patients in each cell of contingency tables is then $\bar{n}$ = 33.

**Figure 2:**
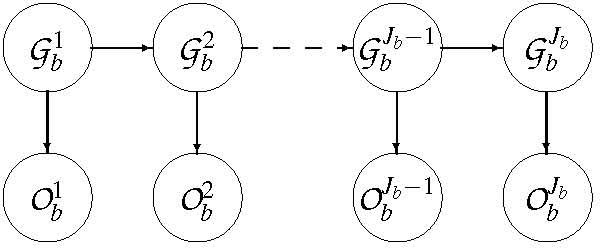
Error and LD model of bin *b*.

An error model (Figure 2) is introduced linking observed genotypes $O_{b}^{j}$ with real ones ($O_{b}^{j}$ ∈ {*aa*, *Aa*, *AA*, ∅}, where ∅ means that the observed genotype is missing):

(14) $$P(O_{b}^{j}|{\left(G_{b}^{l}\right)}_{l\in [1,J_{b}]})=P(O_{b}^{j}|G_{b}^{j})$$

Since $G_{b}^{j}$ are hidden variables, estimation of a priori probabilities of $P(G_{b}^{j}|G_{b}^{j-1})$ and $P(O_{b}^{j}|G_{b}^{j})$ is not straightforward. Usual strategy is to use an Expectation-Maximization (E.-M.) algorithm to infer the state of hidden variables. However, it is not required in order to assess bin associations. Therefore, an alternative strategy is developed. $P(G_{b}^{j}|G_{b}^{j-1})$ and $P(G_{b}^{1})$ are estimated through the removal of patients with missing genotypes:

(15) $$\hat{P}(G_{b}^{j}|G_{b}^{j-1})=\frac{n(O_{b}^{j},O_{b}^{j-1})+C}{n(⊕,O_{b}^{j-1})-n_{\emptyset }+mC}$$

Where n_∅ _is the number of patients with either $O_{b}^{j}$ or $O_{b}^{j-1}$ missing and and *m *is the number of cells. To obtain more regular estimates, a constant is added to all cell counts. It is a Dirichlet prior on parameters. This constant is chosen to be *C *= *α*_0_$\bar{n}$, where *α*_0 _is the chosen error rate and $\bar{n}$ is the mean number of individuals per cell. This constant means that uncertainty on low cell counts is high, not only because of randomness, but also because of genotyping errors.

On the other hand, given the previously developed structure of errors, the following model of $P(O_{b}^{j}|G_{b}^{j})$ is chosen:

(16) $$P(O_{b}^{j}|G_{b}^{j})=\left(\begin{array}{rrrr} O_{b}^{j}\backslash G_{b}^{j} & aa & Aa & AA\\ aa & \begin{array}{r} 1-\beta \\ 1-\alpha \end{array} & \begin{array}{r} 1-2\beta \\ \alpha \end{array} & 0\\ Aa & \begin{array}{r} 1-\beta \\ \alpha \end{array} & \begin{array}{r} 1-2\beta \\ 1-2\alpha \end{array} & \begin{array}{r} 1-\beta \\ \alpha \end{array}\\ AA & 0 & \begin{array}{r} 1-2\beta \\ \alpha \end{array} & \begin{array}{r} 1-\beta \\ 1-\alpha \end{array}\\ \emptyset & \beta & 2\beta & \beta \end{array}\right)$$

The missing rate *β *is estimated for each marker through the resolution of the non-linear system drawn from the preceding model. The maximum error rate *α*_0 _is estimated during external comparison of *Affy. technology *and other technologies. In this study, the error rate is chosen to be *α*_0 _= 0.05. The error rate is *α *= min(*α*_0_, *P*($O_{b}^{j}$ = *Aa*)/(1 - *P *($O_{b}^{j}$ = ∅))) in order that the system always have a solution for *β*.

#### Likelihood computation

With the current model, the likelihood of a patient is the sum of the likelihoods over all possible combinations of real genotypes:

(17) $$L_{1}(i_{b})=p\left(o_{b}^{j}(i)\right)=\sum_{g_{b}^{j}\in [1,\#(G_{b}^{j})]}\left(\prod_{j>1}p\left(o_{b}^{j}(i)|g_{b}^{j}\right)p\left(g_{b}^{j}(i)|g_{b}^{j-1}(i)\right)p(o_{b}^{1}(i)|g_{b}^{1})p(g_{b}^{1})\right)$$

**Figure 3:**
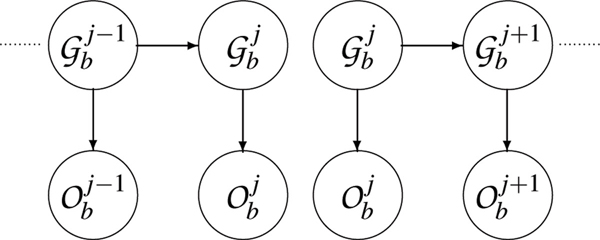
Simplified model of two-marker likelihood computation.

This is a computation in $O\left(\prod \#(G_{b}^{j})\right)\sim O\left(3^{J_{b}}\right)$. Some approximations in the model are required to obtain computations linear with the number of markers. The following one is based on two-marker sliding windows and corresponds to the model of Figure 3:

(18) $$L_{2}(i_{b})=\prod_{j\geq 2}\sum_{g_{b}^{j-1},g_{b}^{j}}\left(p\left(o_{b}^{j}(i)|g_{b}^{j}\right)p\left(g_{b}^{j}j,g_{b}^{j-1}\right)p(o_{b}^{j-1}(i)|g_{b}^{j-1})\right)$$

This equation considers information coming from two neighbor markers together. Compared to the full model, information flow is limited to pair of markers. The likelihood could be falsely increased in this extreme situation: suppose that a missing genotype is inferred *aa *from its left neighbor and *AA *from its right neighbor, the merging of this two inferences would results in a contradiction and thus a low resulting likelihood. On the contrary, the approximated likelihood does not detect this contradiction and is falsely increased. This likelihood is named thereafter "two-marker" likelihood.

Simplifying further leads to consider markers one by one. There is no model of linkage disequilibrium anymore, but noise is reduced as cells are better filled. This likelihood is named thereafter "naive likelihood" because it corresponds to a naive Bayesian model:

(19) $$L_{3}(i_{b})=\prod_{j}\sum_{g_{b}^{j}}p(o_{b}^{j}(i)|g_{b}^{j})p(g_{b}^{j})$$

## Results

The method has been applied to each of the three collections *A*, *B*, *C *(Table 1) as well as to the three collections at once (*ABC*), considering the collection of origins as a co-variable. The overall computation time is about 10 days on a single processor.

The pre-processing filters discard around 20% of SNP: for collection *A *(resp. *B *and *C*), out of 112 463 SNP, 84 430 (resp. 93 548 and 86 652) SNP remains. If all SNP satisfied the Hardy-Weinberg equilibrium, 2 249 SNP are expected to be discarded. 9 422 were for collection *A*. It can be explained (*i*) by artifacts of DM calling algorithm which has a higher error rate on heterozygotic genotypes (*ii*) by deviations from the assumptions underlying this theoretical equilibrium. The bin partioning algorithm divides the genome into 19 556 gene bins and 1 993 desert bins. Out of these 21 549 bins, only 11 264 (52%) contain one SNP or more after pre-processing in at least one collection and are considered for further analysis. Before pre-processing, out of 12 512 SNP with one bin or more, 2 781 have only one SNP, and 2 188 bins 10 SNP or more. The maximum is 210.

**Figure 4:**
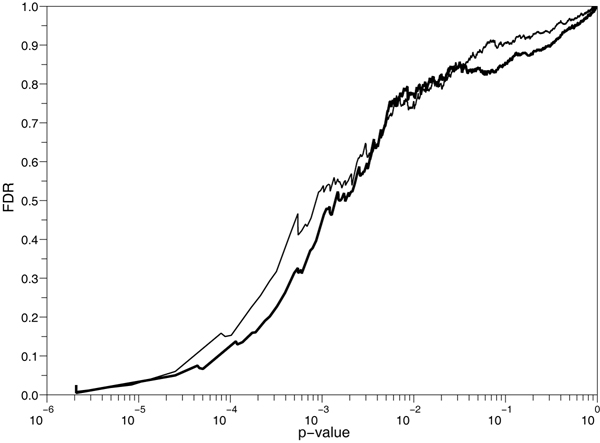
**FDR versus *p*-values of bins sorted in increasing order for the three collections design (*ABC*)**. Thick line: two-marker likelihood *L*_2_, thin line: naive *L*_3_.

**Figure 5:**
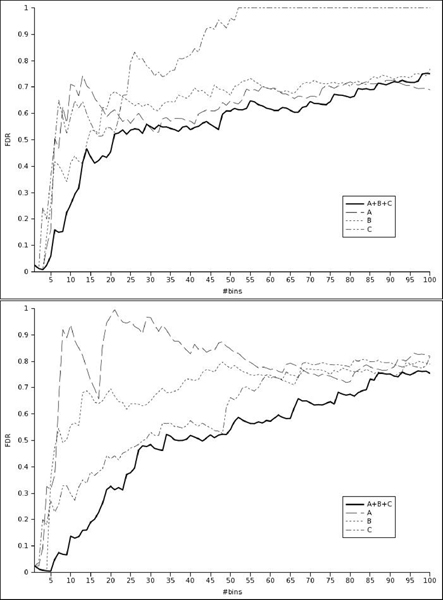
**FDR versus number of bins selected using *L*_3 _naive likelihood (top) and *L*_2 _two-marker likelihood (bottom)**. *A*: solid, *B*: dash, *C*: dash dot, *ABC*: thick.

Figure 4 shows the FDR plotted against *p*-values computed using the two-marker *L*_2 _or the naive *L*_3 _likelihood for the three collection design. Two-marker FDR remains below naive FDR until a *p*-value level of 0.01 and both increase slowly towards 1. FDR against the number of selected SNP plots are detailed by collection in Figure 5. As observed in other studies [13], the FDR is not monotonous with the *p*-value. The oscillations are less important for the three collection design, maybe because of the three time increase of sample size. With a FDR threshold of 5%, only between 2 and 6 bins are selected depending on the collections and likelihood considered (Table 2, top). Most of them are located, in the Major Histocompatibility Complex (MHC) region, mainly in the class III subregion. The class II subregion is known to be associated with MS [14]. The three collection design selects more associated bins than one collection designs, independently on the likelihood. Results with a less stringent FDR threshold of 50% (Table 2, middle) shows a greater power of *L*_2 _over *L*_3 _for the three collection design. However, FDR is misleading in this study because the MHC region is known to be associated with MS. It leads to an overestimation of the FDR at which bins outside of this region are selected. It contains 12 of the 33 bins selected by *L*_2 _on the three collection design. As a result, only 10 and not 21 bins are selected (Table 2, bottom).

**Table 2:** Associated bins at FDR 5% threshold (top), at FDR 50% threshold before (middle) and after exclusion of MHC region bins (bottom). *A*, *B*, *C*, *ABC*: collection designs, *L*_2_: two-marker likelihood, *L*_3_: naive likelihood.

*FDR 5% with MHC*	*L*_3_	*L*_2_
*A*	3	2

*B*	3	6

*C*	2	2

*ABC*	4	6

		

*FDR 50% with MHC*	*L*_3_	*L*_2_

*A*	6	6

*B*	14	7

*C*	6	28

*ABC*	20	33

		

*FDR 50% w/o MHC*	*L*_3_	*L*_2_

*A*	2	0

*B*	1	1

*C*	0	0

*ABC*	8	10

## Discussion

We have developed a new method to practically analyze genome-wide association studies data. Our algorithm is based on a bin partitioning of the genome, takes advantage of studying several collections simultaneously, takes into account genotyping errors and local genomic structure (LD), and handles the multiple testing problem through FDR estimation while staying computationally tractable. The method has been applied to analyze three association studies in Multiple Sclerosis.

The FDR threshold is chosen according to the desired application. To conduct expensive further experiments with putatively associated genes, a very low rate of false-positives is required. A FDR threshold of 5% seems reasonable. On the contrary, if one wants to minimize the false-negative rate, a FDR of 50% is acceptable.

Applying the method to experimental genome-wide association data on three collections permits (*i*) to assess the algorithm and evaluate the different parameters and design and (*ii*) to identify genes potentially associated to Multiple Sclerosis. We have evidenced that the three collection design outperforms the one-study design in terms of expected number of true-positives, despite differences between the studied collections, especially on the severity of the disease. Furthermore, with this three collection design, the two-marker likelihood *L*_2 _seems to be more efficient thanks to the additional information used. With this configuration, a FDR threshold of 5% gives 6 associated bins. Four of them are located in the MHC region, known to be linked to Multiple Sclerosis [14]. It is a validation of the method. The two others are bins containing olfactory receptor genes *OR2T2 *and *OR4A47*. The biological meaning of such association is unclear but the extended MHC regions contain many other olfactory genes [14] and olfactory dysfunction has already been reported in Multiple Sclerosis [15]. At FDR threshold of 50% and after exclusion of bins from MHC, the method selects ten bins. They open the perspective of insights to explain Multiple Sclerosis.

## Competing interests

The authors declare that they have no competing interests.

## Authors' contributions

NO, FK and JW conceived and designed the model. NO, KF, ML and GM wrote the analysis tool. NO, FK and JW wrote the manuscript.
